# Kinetics of glucose-6-phosphate dehydrogenase (G6PD) activity during *Plasmodium vivax* infection: implications for early radical malaria treatment

**DOI:** 10.1186/s12936-024-04973-4

**Published:** 2024-05-09

**Authors:** Laureen Dahuron, Juste Goungounga, Moustapha Drame, Maylis Douine, Mathieu Nacher, Théo Blaise, Emilie Mosnier, Lise Musset, Marie Fouillet, Félix Djossou, Loïc Epelboin

**Affiliations:** 1 Infectious and Tropical Diseases Department, Centre Hospitalier de Cayenne Andrée Rosemon, French Guiana, Cayenne, France; 2 Université de Rennes, EHESP, CNRS, Inserm, Arènes-UMR 6051, RSMS-U 1309, 35000 Rennes, France; 3 Département METIS, Écoles des Hautes Études en Santé Publique, Rennes, France; 4 Department of Clinical Research and Innovation, University Hospital of Martinique, Fort‐de‐France, Martinique, France; 5 Centre d’Investigation Clinique Antilles-Guyane (CIC Inserm 1424), Centre Hospitalier de Cayenne Andrée Rosemon, French Guiana, Cayenne, France; 6 Sciences Economiques et Sociales de la Santé et Traitement de l’Information Médicale, UMR1252, Aix Marseille Univ, INSERM, IRD, SESSTIM, Marseille, France; 7 ANRS MIE Cambodian Site, University of Health and Science, Phnom Penh, Cambodia; 8 Laboratoire de parasitologie, Centre Nationale de Référence du Paludisme, World Health Organization Collaborating Centre for Surveillance of Antimalarial Drug Resistance, Institut Pasteur de la Guyane, French Guiana, Cayenne, France

**Keywords:** *Plasmodium vivax*, G6PD/Glucose-6-phosphase dehydrogenase, Malaria, Primaquine, French Guiana

## Abstract

**Background:**

*Plasmodium vivax* relapses due to dormant liver hypnozoites can be prevented with primaquine. However, the dose must be adjusted in individuals with glucose-6-phosphate-dehydrogenase (G6PD) deficiency. In French Guiana, assessment of G6PD activity is typically delayed until day (D)14 to avoid the risk if misclassification. This study assessed the kinetics of G6PD activity throughout *P. vivax* infection to inform the timing of treatment.

**Methods:**

For this retrospective monocentric study, data on G6PD activity between D1 and D28 after treatment initiation with chloroquine or artemisinin-based combination therapy were collected for patients followed at Cayenne Hospital, French Guiana, between January 2018 and December 2020. Patients were divided into three groups based on the number of available G6PD activity assessments: (i) at least two measurements during the *P. vivax* malaria infection; (ii) two measurements: one during the current infection and one previously; (iii) only one measurement during the malaria infection.

**Results:**

In total, 210 patients were included (80, 20 and 110 in groups 1, 2 and 3, respectively). Data from group 1 showed that G6PD activity remained stable in each patient over time (D1, D3, D7, D14, D21, D28). None of the patients with normal G6PD activity during the initial phase (D1–D3) of the malaria episode (n = 44) was categorized as G6PD-deficient at D14. Patients with G6PD activity < 80% at D1 or D3 showed normal activity at D14. Sex and reticulocyte count were statistically associated with G6PD activity variation. In the whole sample (n = 210), no patient had severe G6PD deficiency (< 10%) and only three between 10 and 30%, giving a G6PD deficiency prevalence of 1.4%. Among the 100 patients from group 1 and 2, 30 patients (26.5%) were lost to follow-up before primaquine initiation.

**Conclusions:**

In patients treated for *P. vivax* infection, G6PD activity did not vary over time. Therefore, G6PD activity on D1 instead of D14 could be used for primaquine dose-adjustment. This could allow earlier radical treatment with primaquine, that could have a public health impact by decreasing early recurrences and patients lost to follow-up before primaquine initiation. This hypothesis needs to be confirmed in larger prospective studies.

**Supplementary Information:**

The online version contains supplementary material available at 10.1186/s12936-024-04973-4.

## Background

According to the 2022 World Health Organization (WHO) report, malaria still represents a global health issue. In 2021, the WHO estimated 247 million cases of malaria in 86 endemic countries [1]. However, in the Americas, malaria cases decreased by 60% (from 1.5 million to 0.6 million) between 2000 and 2021 and mortality was reduced by 64%. In this region, 71.5% of malaria infections are caused by *Plasmodium vivax*. This *Plasmodium* produces hypnozoites, which are dormant forms of the parasite that can cause relapses [1]. Relapses may occur weeks to months after the initial infection hampering *P. vivax* elimination. Therefore, the WHO suggests combining drugs to eliminate blood forms (chloroquine or artemisinin-based combinations) and drugs to eliminate hepatic hypnozoites (8-aminoquinolines: primaquine or tafenoquine). This is known as radical treatment. However, the currently available drugs to eliminate hypnozoites (8-aminoquinolines), can cause haemolysis in patients with glucose-6-phosphate dehydrogenase (G6PD) deficiency [2]. To avoid iatrogenic haemolysis, the WHO recommends systematically screening for G6PD deficiency before primaquine initiation in order to adapt the dose and duration of primaquine treatment to the patient's G6PD status. Specifically, patients with G6PD enzyme activity < 30% receive primaquine (0.75 mg/kg body weight) once per week for 8 weeks, instead of the standard regimen (0.5 mg/kg/day for 14 days) [3]. In 2018 the daily dose of primaquine recommended by the French High Council for Public Health (HCSP) for French Guiana was 0.5 mg/kg/day (i.e., 30 mg/day for an adult weighing 60 kg), in one or two doses per day, for 14 days [4].

G6PD deficiency is an X-linked recessive genetic disorder, with more than 230 variants identified worldwide [5, 6]. It is the most common enzymopathy in humans, and it concerns over 400 million people worldwide [5, 7]. Haemolysis intensity depends on variant: severe for Mediterranean B- and A- variants (< 1% of the normal activity), and mild for the African A- variant (10–15% of the normal activity). In Latin America, G6PD deficiency prevalence is low (< 2%) and the African A- variant is predominant. In French Guiana, a multi-ethnic territory located in the North of South America between Suriname and Brazil, the exact prevalence of G6PD deficiency is unknown, but the A- variant might be predominant [8–11].

G6PD screening is recommended in French Guiana before radical treatment of *P. vivax* malaria. The HCSP, highlighted the risk of underestimating G6PD levels if G6PD screening is performed early during an acute malaria episode (i.e., before day 14). [4, 12] Indeed, reticulocytes have higher G6PD activity than mature red blood cells and the initial haemolysis stimulates erythropoiesis (increased reticulocyte count), which could result in overestimating G6PD level [13–16]. In clinical practice, primaquine can be initiated immediately if G6PD deficiency has been already ruled out, otherwise G6PD activity is tested on day 14. Then, compassionate use authorization is requested on day 21 (upon reception of the G6PD activity results), and primaquine is dispensed from day 21, thus increasing the risk of patients being lost to follow-up. To avoid this, G6PD activity is measured earlier at the Infectious and Tropical Diseases Department of the Cayenne hospital, French Guiana, based on the hypothesis that G6PD activity during a *P. vivax* malaria episode is stable over time and that the malaria-related haemolysis does not hide a G6PD deficiency. Indeed, the early initiation of radical treatment would reduce relapses, morbidity, anaemia, hospitalizations, and the number of patients lost to follow-up. It could also decrease inter-individual transmission and the economic burden of *P. vivax* malaria. However, experimental data are needed to support this pragmatic approach.

Therefore, the main objective of this study was to analyse the variations in G6PD activity during *P. vivax* malaria between day-1 and day-28 after a treatment initiation (chloroquine or artemisinin-based combinations) in order to determine whether early measurements reliably ascertain the absence of G6PD deficiency. The secondary objectives were to identify factors that influence G6PD activity during *P. vivax* infection; to estimate the prevalence of G6PD deficiency in patients treated for *P. vivax* infection in French Guiana and to evaluate the percentage of patients lost to follow-up before primaquine treatment.

## Methods

### Study design and population

For this retrospective monocentric study carried out in French Guiana, patients with *P. vivax* infection diagnosed between January 1, 2018 and December 31, 2020 were included if they had been treated with chloroquine or ACT and had at least one G6PD measurement. *Plasmodium vivax* infection diagnosis was based on the observation of asexual parasites on thin and thick blood films and was performed at the Parasitology Laboratory of Cayenne hospital, French Guiana. Patients with mixed infection (*P. vivax* and another plasmodial species) were excluded. The medical records of the included patients (inpatients and outpatients at the Infectious Diseases Department of Cayenne hospital were reviewed. Data on primaquine delivery were obtained from the Cayenne hospital pharmacy.

### Data collection and analysis

Medical records were reviewed retrospectively. Demographic data, comorbidities, and laboratory data (day 1, 3, 7, 14, 21, 28) were collected. Day 1 was the first day of treatment with chloroquine or artemisinin-based combination. For G6PD activity quantification, blood samples were shipped at 4 °C to mainland France (Paris) for testing at the Biomnis Laboratory, Ivry-sur-Seine. G6PD activity (U/g Hb) was measured using an enzyme-linked immunosorbent assay based on the evaluation of absorbance at 340 nm given by NADPH formation at 37 °C (Pointe Scientific—COBAS Roche^®^, Canton, MI, États-Unis). Pyruvate kinase and hexokinase assays were not routinely performed. G6PD activities < 10%, < 30%, and > 80% corresponded to an assay result < 1 U/g Hb, < 3 U/g Hg, and > 8 U/g Hb G6PD activity, respectively. Patients were classified using the following thresholds: G6PD < 10% (severe deficiency), 10% < G6PD < 30% (deficiency), 30% < G6PD < 80% (intermediate), and G6PD ≥ 80% (normal). Data on reticulocyte and platelet count (G/L), and hemoglobin concentration (g/dL) measured using a SYSMEX XN 10 automated hematology analyzer, and on creatinine (µmol/L), bilirubin (µmol/L) lactate dehydrogenase (LDH—IU/L) and haptoglobin concentration (mg/L) measured using COBAS c501 analyzer, were extracted from the medical records.

**Fig. 1:**
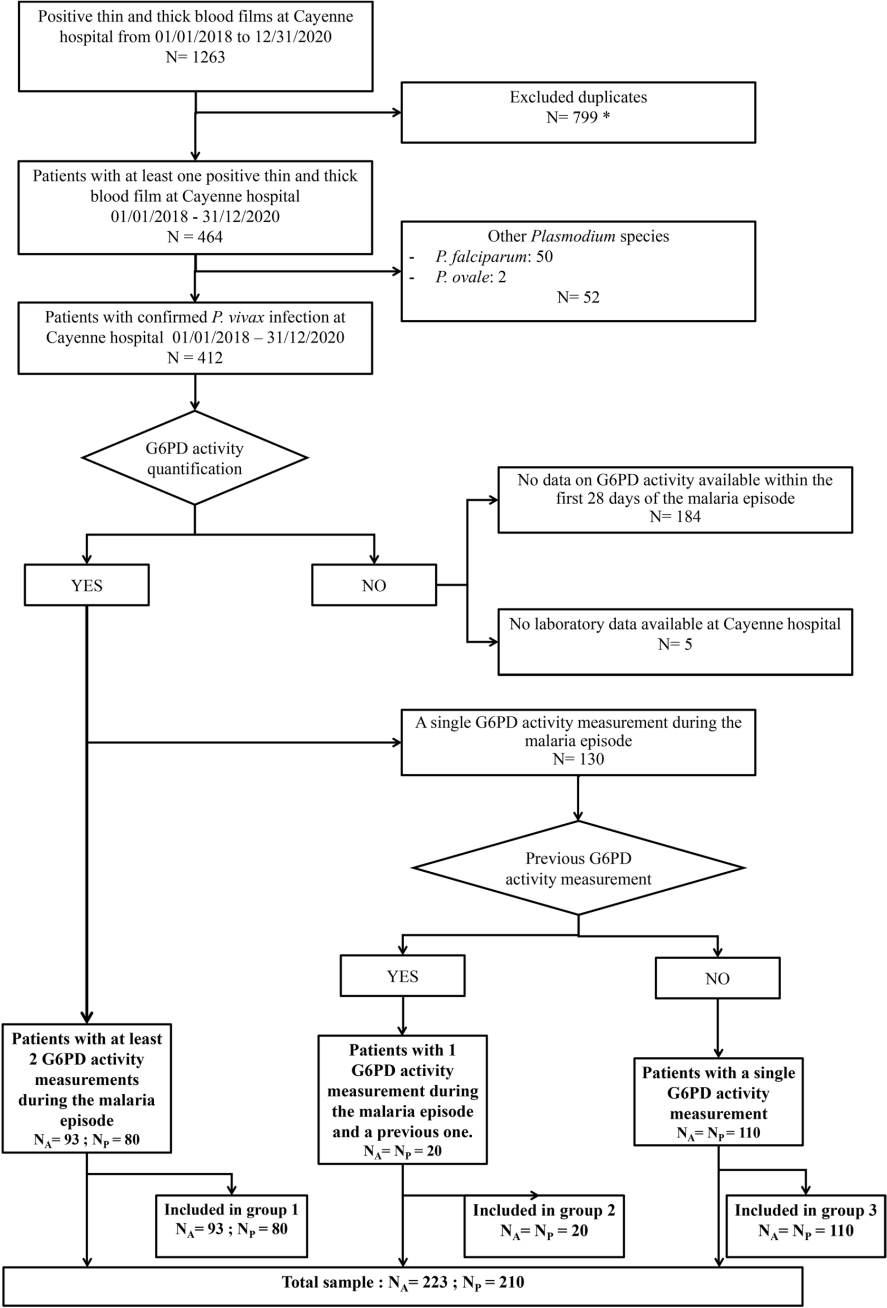
Study flowchart. N_A_ = Number of *Plasmodium vivax* attacks; N_P_ = Number of patients (one or more malaria episodes). (Asterisk) Duplicates: multiple results for the same malaria episode. *G6PD* glucose-6-phosphate dehydrogenase

Patients were divided into three groups, in function of the number of available G6PD activity assays results: group 1 (patients who had at least two G6PD activity measurements during the study period), group 2 (one G6PD activity measurement during the study period and one previous measurement), and group 3 (patients with only one G6PD activity measurement during the study period). The total sample included all patients with at least one G6PD activity measurement (Fig. 1).

As three patients experienced more than one malaria episode between January 2018 and December 2020, so the number of malaria attack (N_A_) differs from the total number of patients (N_P_).

### Statistical analyses

Data processing and statistical analysis were performed with the R software (version R-4.2.). Due to missing data, the analysis was limited to complete data. Fixed effects related to clinical and laboratory covariates and random effects related to G6PD activity in each patient and over time were retrospectively tested. This method was chosen because the laboratory variables were measured at repeated intervals and their distribution was heterogeneous. (Supplement 1). This modeling strategy was used to analyse G6PD activity variability and reticulocyte count over time in a post hoc analysis. A type III analysis of variance (mixed ANOVA) was then performed to assess G6PD activity variability over time. Multivariate analysis was performed by backward selection of covariates potentially associated with G6PD activity, based on the Akaike information criterion (AIC). The Restricted Maximum Likelihood Estimation Criterion (REMLC) was used to identify the estimated parameter of the linear parsimonious random-effects model [17]. The Wald test was used to measure the significance of the coefficients (0.05 level of significance).

### Ethical concerns

This was a non-interventional study not involving human persons and using data collected as part of routine care. This research was carried out in compliance with the French Data Protection Act of January 6, 1978, as amended, and article No. 2018-493 of June 20 2018 on the protection of personal data and the General Data Protection Regulation (EU) 2016/679 of the European Parliament and of the Council of April 27, 2016.

Data were collected in accordance with the reference methodology MR004 of the Commission Nationale de l'Informatique et des Libertés (CNIL), for which the Centre Hospitalier de Cayenne has signed a compliance agreement. Patients came from the Infectious Diseases Department of Cayenne Hospital, and all analyses were carried out by Infectious Diseases Department staff. This is an internal research study in the sense of the CNIL on which collective information was given to the included patients, with the possibility of expressing their opposition to the collection of their data. The study was registered with the hospital data protection officer. Access to data for research purposes took place on October 2, 2021. Data collected from medical records were anonymized by code and year of birth. Only information strictly necessary for the research was collected.

## Results

### Patients’ baseline characteristics

During the study period, 80 patients were included in group 1, 20 patients in group 2, and 110 patients in group 3 (Fig. 1), thus 210 patients in total. In group 1 and 2 together (n = 100 patients with two G6PD activity measurements), 56 patients were men and 44 women (male to female sex ratio = 1.3), and their median age was 31 years (interquartile Range—IQR 19–46.8). Seventy patients (70%) had no major comorbidity, forty-five patients were born in French Guiana (45%), and 42 were born in Brazil (42%) (Table 1). Moreover, 62 patients (55%) had a previous history of malaria, and only five patients (5.4%) had features of severe malaria (Table 2). Thirty patients (27%) were lost to follow-up before primaquine initiation (Table 2), and the interval between chloroquine initiation and primaquine initiation ranged from 3 to 316 days (median interval 28 days IQR: 20.5–49.5). Forty patients (35.4%) had a relapse among whom, 10 before primaquine initiation (lost to follow-up). The median time from malaria attack to relapse was 63 days (IQR 40.3–92.3) and ranged from 20 to 490 days. (Table 3).

**Table 1:** Patients’ baseline characteristics

Parameter	Group 1 n = 80 n (%)	Group 2 n = 20 n (%)	Group 1 + 2 n = 100
Sex			
Male	43 (54)	13 (65)	56
Female	37 (46)	7 (35)	44
Age (years): median (IQR)/range	31 (19–50)/0–77	26 (19–42)/4–49	31 (19–47)/0–77
< 20 years	21 (26)	7 (35)	28
20–39 years	30 (38)	7 (35)	37
40–59 years	20 (25)	6 (30)	26
60–79 years	9 (11)	0 (0)	9
> 80 years	0 (0)	0 (0)	0
At-risk groups			
Military	6 (78)	1 (5)	7
Gold mining	12 (15)	2 (10)	14
Comorbidities			
Pregnancy	1 (1)	2 (10)	3
High blood pressure^a^	6 (78)	0 (0)	6
Diabetes mellitus	3 (4)	1 (5)	4
Overweight^b^	7 (9)	2 (10)	9
HIV	1 (1)	0 (0)	1
Immunosuppressive treatment	0 (0)	0 (0)	0
Cancer history	3 (4)	0 (0)	3
Psychiatric history	1 (1)	2 (10)	3
Kidney failure	0 (0)	1 (5)	1
Country of birth			
France^c^	35 (44)	10 (50)	45
Brazil	36 (45)	6 (30)	42
Other	6 (8)	2 (10)	8
Unknown	3 (4)	2 (10)	5

*IQR* interquartile ranges, *HIV* human immunodeficiency virus

^a^High blood pressure: blood pressure ≥ 140–90 mmHg on two different days

^b^Overweight: BMI > 25

^c^Including French Guiana

**Table 2:** Malaria episode characteristics (discrete variables)

Parameter	Group 1 n = 93^a^ n (%)	Group 2 n = 20 n (%)	Group 1 + 2 n = 113
Malaria history	52 (56)	10 (50)	62 (55)
≥ 3 malaria episodes	7 (8)	10 (50)	17 (15)
Number of relapses	30 (32)	10 (1)	40 (35)
Relapse within 90 days	23 (25)	5 (0.3)	28 (25)
Hospitalization for malaria	38 (41)	3 (15)	41 (36)
Severity criteria	5 (5)	0 (0)	5 (4)
Blood transfusion	1 (1)	0 (0)	1 (1)
Lost to follow-up before primaquine initiation	21 (23)	9 (45)	30 (27)
Number of relapses in patients lost to follow-up	6	6	10
Followed at a CDPS	11 (12)	13 (65)	24 (21)
Chloroquine	89 (96)	19 (95)	108 (96)
ACT	4 (4)	1 (5)	5 (4)

*ACT* Artemisinin-based Combination Therapy, *CDPS* delocalized center of treatment and prevention

^a^N_A_: malaria episodes during the study period (some patients had more than one episode)

**Table 3:** Malaria episode characteristics (continuous variables)

	Group 1 n = 93^a^		Group 2 n = 20		Group 1 + 2 n = 113	
	Median (IQR)	Range	Median (IQR)	Range	Median (IQR)	Range
*P. vivax* parasitemia (%)	0.16; (0.04–0.30)	0.001–2	0.11; (0.04–0.37)	0.01–1	0.15; (0.04–0.34)	0.001–2
Interval between chloroquine and primaquine (days)	27; (20–39)	3–290	72; (49–149)	28–316	28; (21–50)	3–316
Body weight (kg)	62; (50–79.25)	8.4–150	65; (58–76)	19–104	65; (50–79)	8.4–150
Primaquine dose (mg/kg)	0.44; (0.38–0.54)	0.15–0.67	0.46; (0.40–0.48)	0.29–0.53	0.44; (0.38–0.52)	0.15–0.67
Time to relapse (days)	63; (37–86)	20–331	78; (56–101)	44–43,788	63; (40–92)	20–490

*IQR* interquartile range

^a^N_A_: malaria episodes during the study period (some patients had more than one episode)

**Fig. 2:**
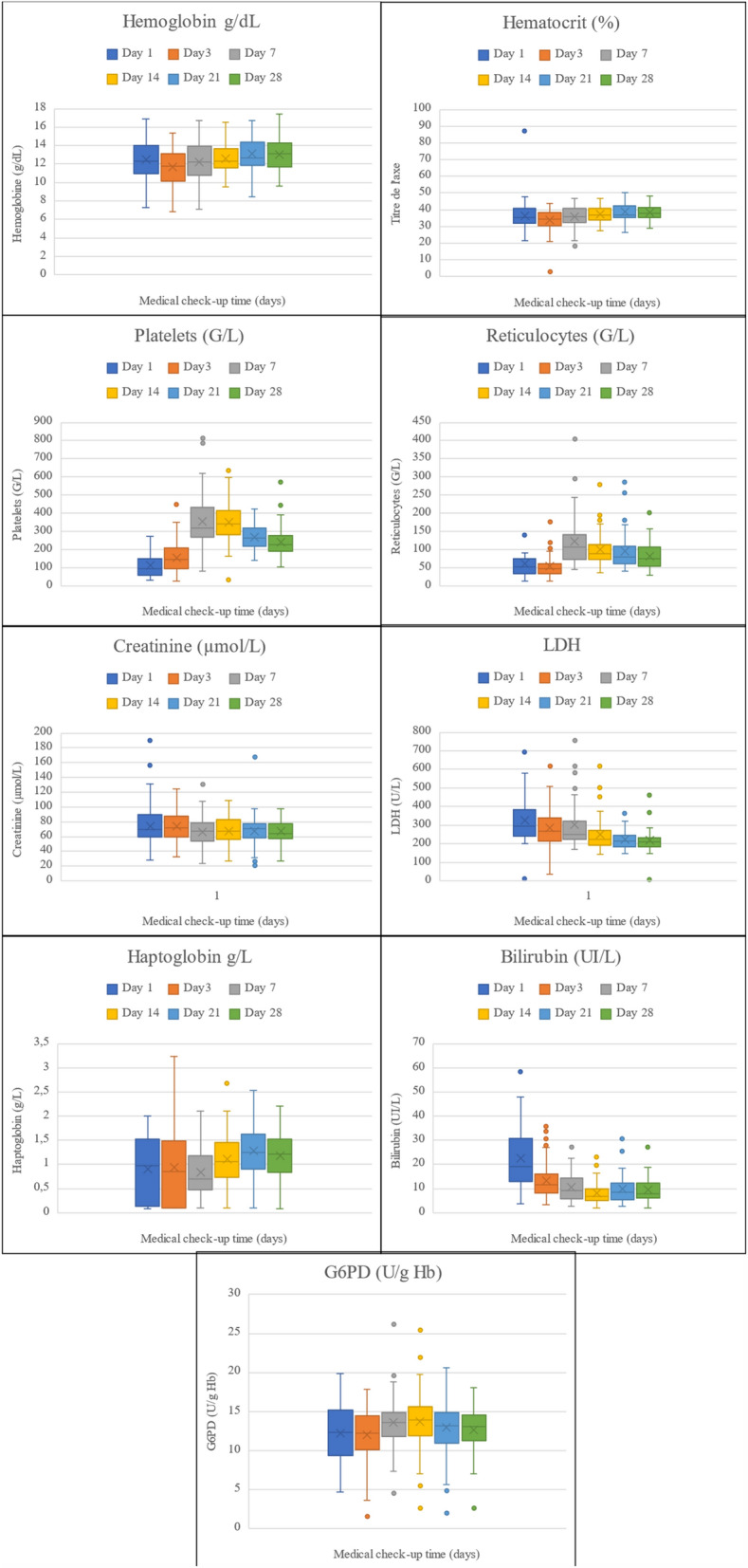
Box plots (median, interquartile range, full range) showing the distribution of the indicated laboratory parameter values in group 1 (N_A_ = 93) at different time points. The line indicates the median, crosses indicate the mean. D, day after treatment (chloroquine or artemisinin-based combination) initiation

Figure 2 shows the changes of laboratory variables in group 1 (the only group for whom these data were available) during *P. vivax* infection LDH and bilirubin concentration decreased over time, while haemoglobin, platelets, reticulocytes and haptoglobin started to increase from day 7. Conversely, haematocrit, G6PD activity and creatinine remained relatively stable.

### G6PD activity kinetics

Supplement 2 shows the individual variability of G6PD activity in group 1 at the different time points after treatment initiation (chloroquine or ACT). This variability was higher in women than in men (Supplement 3, Supplement 4). The mixed ANOVA showed a significant difference in the mean G6PD activity between day 1 and day 28 (p value = 0.004), and in reticulocyte count (p value < 0.001).

**Fig. 3:**
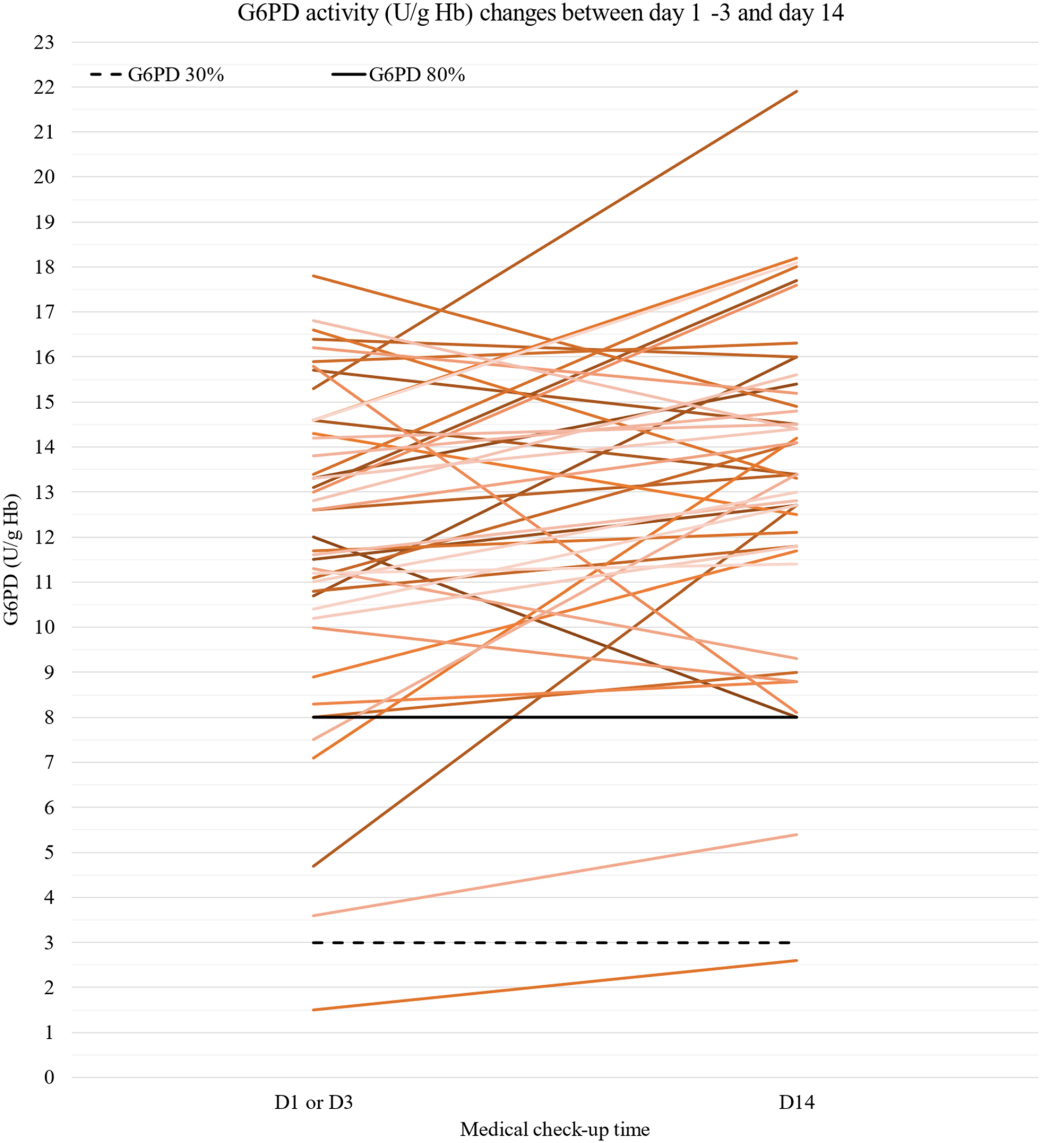
G6PD activity (U/g Hb) changes in the 44 patients from group 1 with G6PD activity measurements at day 1 or day 3 and at day 14 after treatment initiation (chloroquine or artemisinin-based combination). Dashed black line and black line: 30% and 80% of G6PD activity, respectively. *D* day

In group 1 (n = 80), G6PD activity was measured at day 1 or day 3 and at day 14 only in 44 patients. In none of them, G6PD assay was falsely normal at day 1 or day 3. Conversely, in four patients with G6PD activity < 80% at day 1-day 3, the activity was back to normal (> 80%) at day 14 (Fig. 3).

### Factors modifying G6PD activity

**Fig. 4:**
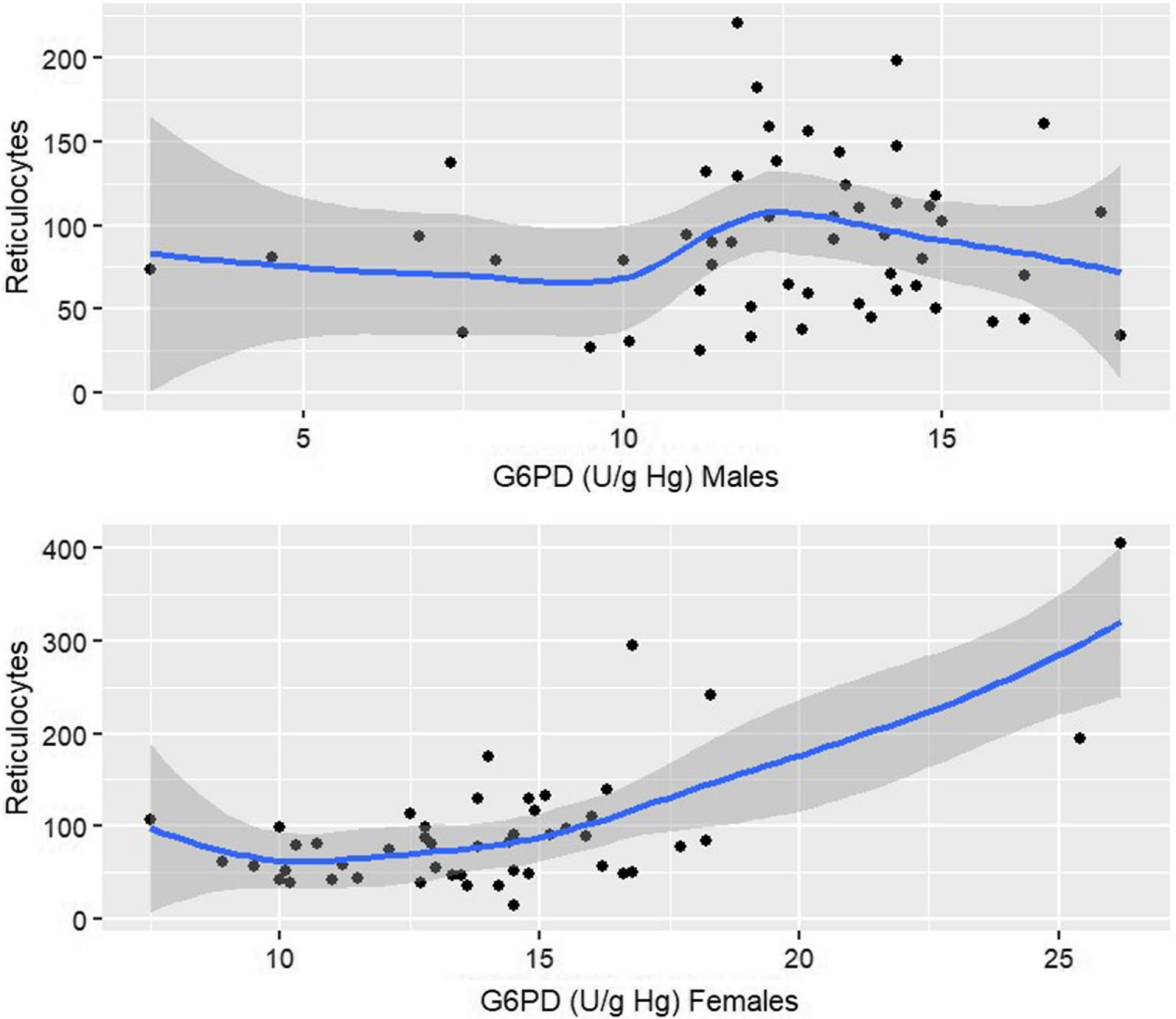
Scatterplot (black points) and smoothing line (blue line and its 95% confidence intervals) showing the relationship between G6PD activity and reticulocyte count by sex group. Reticulocyte count (G/L), G6PD (U/g Hb)

Clinical and demographic characteristics were included in the multivariate analysis that took into account G6PD activity in each patient and its variability over time. The best model was the one that did not take into account G6PD activity over time (model 1—AIC 52.820). Among the demographic covariates, only sex was identified as a factor associated with the G6PD activity kinetics (β = 0.181 [0.044; 0.318]; p value = 0.010) (Table 4). G6PD activity was higher and more variable in women than in men. G6PD activity was higher in women compared with men when controlling reticulocyte count (0.18 times higher) (Table 4). Figure 4 suggests that the correlation between G6PD and reticulocyte count was log-linear in women but not in men.

**Table 4:** Multivariate linear mixed effect models with clinical, demographic and laboratory variables to identify the determinants of G6PD activity

Effects	Variables/terms	Estimate	95% CI lower	95% CI higher	P value	P value (days)
Model 1						
Fixed effects						
	(Intercept)	1.998	1.673	2.323	< 0.001	
	Sex	0.181	0.044	0.318	0.010	
	ln (reticulocytes)	0.101	0.030	0.173	0.006	
Random effects						
	SD_(Intercept)|id	0.263	0.212	0.316		
	Sigma	0.200	0.177	0.226		
	AIC	52.820				
Model 2						
Fixed effects						
	(Intercept)	2.014	1.610	2.417	< 0.001	0.690
	Day3	0.036	− 0.112	0.185		
	Day7	− 0.065	− 0.200	0.071		
	Day14	0.049	− 0.054	0.152		
	Day21	0.019	− 0.069	0.107		
	Day28	0.034	− 0.037	0.106		
	Sex	0.184	0.046	0.321	0.009	
	ln (reticulocytes)	0.094	0.003	0.186	0.043	
Random effects						
	SD_(Intercept)|id	0.263	0.212	0.316		
	Sigma	0.201	0.175	0.223		
	AIC	59.760				
Model 3						
Fixed effects						
	(Intercept)	2.508	2.430	2.586	< 0.001	0.272
	Day3	0.076	− 0.067	0.219		
	Day7	− 0.097	− 0.229	0.035		
	Day14	0.038	− 0.066	0.142		
	Day21	0.050	− 0.034	0.135		
	Day28	0.008	− 0.061	0.077		
Random effects						
	SD_(Intercept)|id	0.278	0.227	0.337		
	Sigma	0.203	0.177	0.226		
	AIC	67				

Model 1 describes the individual variability in G6PD activity without taking into account its variability over time, with sex and reticulocyte count as co-variables retained from the backward selection procedure. Model 2 describes G6PD activity variability in each patient and over time, with sex and reticulocyte count as co-variables retained from the backward selection procedure. Model 3 describes G6PD activity variability in each patient and over time, without any co-variable

G6PD activity was also associated with reticulocyte count (β = 0.101 [0.030; 0.173]; p value = 0.006) (Table 4) whereas there were no significant variations among the other biological variables.

G6PD activity varied among individuals (SD = 0.263 [0.212; 0.316]). Conversely, in each patient, G6PD activity did not vary over time in the presence of covariates (Model 2, p-value (days) = 0.690), and in the absence of adjustment for sex and reticulocyte count (Model 3, p-value (days) = 0.272) (Table 4, Supplement 5).

### Prevalence of G6PD deficiency

**Fig. 5:**
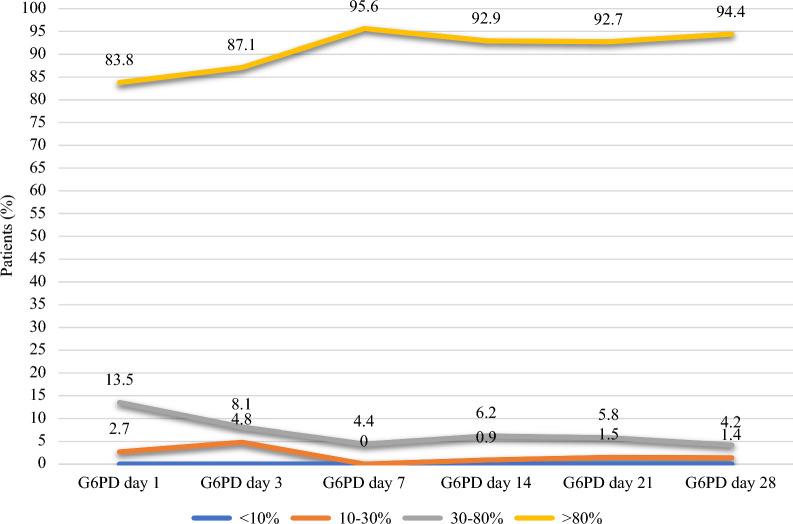
G6PD activity kinetics over time. Prevalence of patients in each G6PD activity category (< 10%, 10–30%, 30–80%, and > 80%). G6PD (U/g Hb). Day 1: n = 37, Day 3: n = 62, Day 7: n = 67, Day 14: 113, Day 21: n = 69, Day 28: n = 71

None of the 210 patients included (all three groups together), had G6PD activity < 10%. Three male patients (1.4%) had activity between 10 and 30%. Results were not different when considering only patients with G6PD measured from at day 7 onwards after treatment (chloroquine or artemisinin-based combination) initiation (n = 202) (Table 5, Supplement 6). Figure 5 shows the prevalence of patient with G6PD deficiency at the different time points after treatment (chloroquine or artemisinin-based combination) initiation.

**Table 5:** Prevalence of G6PD deficiency in patients treated for *P. vivax* infection in French Guiana between January 1, 2018 and December 31, 2020

G6PD activity (%)	Prevalence of G6PD deficiency (%), n = 210^a^	Prevalence of G6PD deficiency from day 7 onwards (%), n = 202^b^	
< 10	0	0	
10–30	3 (1.4)	2 (1)	
30–80	8 (3.8)	6 (3)	
> 80	212 (94.8)	194 (96)	

^a^All patients N_p_ (three groups together)

^b^Only patients with G6PD measured at day 7, day 14, day 21 or day 28

## Discussion

This study showed that G6PD activity level varies among patients with *P. vivax* infection, but not over time in the same person (day 1 to day 28 after chloroquine or ACT initiation). Importantly, none of the patients with normal G6PD activity during the initial phase of the malaria episode (day 1) was categorized as having G6PD deficiency at day 14. Thus, G6PD activity at baseline (day 1) could be used as reference value for ruling out G6PD deficiency and initiating primaquine treatment early during *P. vivax* infection.

These results are consistent with the literature. The study by Taylor et al*.* [15] in Cambodian patients showed that no G6PD-deficient patient was misclassified as having normal G6PD during an acute episode of *P. vivax* malaria. They found that in men with G6PD deficiency, G6PD activity never reached the normal range, and baseline G6PD activity was unaffected by fever.

Furthermore, the present results show a direct link between G6PD activity and reticulocytosis, in agreement with the literature. Indeed, in the same individual, G6PD activity varies in function of the erythrocyte population age. Specifically, G6PD activity peaks in reticulocytes and young red blood cells, and then declines as erythrocytes age [2, 14, 18].

Despite theoretical variations in G6PD activity during haemolysis, the present study showed that in each patient, G6PD activity remained stable during a malaria episode. Several hypotheses can be formulated. First, this study concerned a sample of patients infected by *P. vivax* only, with few severe cases (4%) and low haemolysis [19]. Second, changes in G6PD activity associated with acute haemolysis may differ from one variant to another. The African variant (A-), predominant in South America, causes mild deficiency and mild haemolysis in deficient individuals compared with other variants [8, 10, 20].

Among the 210 patients included in the prevalence analysis, none had G6PD activity < 10% and 1.4% of patients had G6PD activity between 10 and 30%. However, as G6PD deficiency weakens red blood cells and provides protection against malaria [18, 21], the real prevalence of individuals with G6PD deficiency in the general population may have been underestimated [20]. Nevertheless, this finding is consistent with literature data showing a low prevalence of G6PD deficiency in the Americas (< 2%) [10, 22].

Ley et al. [23]*.* measured G6PD activity in patients from Bangladesh, Indonesia, and Ethiopia at the time of malaria infection and 6 to 33 months after the infection. They found that G6PD activity was significantly higher during the malaria episode. They concluded that the G6PD status of an individual is not static and, therefore, the risk of drug-induced haemolysis in malaria cannot be predicted by enzymatic activity far from the acute episode (i.e. during aparasitaemia). In another study in Bangladesh, Ley et al. [24]*,* showed that G6PD activity was higher in individuals with acute malaria than without. Therefore, they suggested that 8-aminoquinoline treatment may be safer than expected in patients with clinical malaria.

The present study has several important limitations. First, its design (retrospective descriptive study) may imply several biases due to missing data. Furthermore, it did not include a control group (i.e. malaria-free controls) to compare changes in G6PD activity in the absence of malaria infection. This is important given the physiological variation in G6PD activity within a single day [25]. Another limitation is the small sample size and particularly, the small percentage of patients with G6PD deficiency. This is explained by the absence of patients with more than one G6PD assays result before 2018 and the drastic decrease in malaria cases in our territory since 2020. Moreover, it would have been important to compare G6PD activity with hexokinase or pyruvate kinase activity. Indeed, G6PD samples are shipped from French Guiana to mainland France and the variable transit times may alter sample quality and affect the results. However, these tests are not routinely performed, and these data were not available. Finally, the usual follow-up of patients did not include a G6PD assay several months after the malaria episode, although it would have been very useful to determine the true basal levels in the included patients [23].

Measuring G6PD activity in the initial phase of a *P. vivax* malaria episode could have a considerable impact in French Guiana. By using the G6PD activity measurement on day 1 as reference value (instead of the one at day 14), radical treatment with 8-aminoquinolines (primaquine or tafenoquine) could be started at least 14 days earlier, around day 3-day 7 instead of day 21. In the case of initial deficiency, G6PD activity quantification could be repeated in the aparasitaemic phase and compared with a pyruvate kinase test to confirm or not the deficiency. In the present study, 30 patients were lost to follow-up before primaquine initiation (i.e. 27% of the 113 patients in groups 1 and 2), a high proportion compared with the literature from neighbouring countries [26, 27]. This is a high percentage in view of the high incidence of *P. vivax* relapses (35% in this study), especially in the absence of radical treatment. Thus, if clinical practice could be modified as proposed, it would be possible to avoid early relapses, reduce the number of patients lost to follow-up before primaquine initiation, and decrease the risk of subsequent relapses. Reducing the incidence of relapses could have an impact on individual morbidity, but also on public health by decreasing the number of hospitalizations, inter-individual transmission, and healthcare costs. However, this proposition requires to be confirmed in a prospective study with a control group to determine the safety of early primaquine introduction.

This change in management could also have an impact on treatment adherence. Khanticul et al*.*[28] showed that 76.2% of patients with *P. vivax* malaria did not adhere to anti-malarial therapy. By starting primaquine earlier, the total follow-up time would be reduced, which could contribute to improve adherence, particularly in remote populations. A marketing authorization for tafenoquine, which is taken as a single dose (versus 14 days for primaquine), would also improve patient adherence and reduce the number of patients lost to follow-up before radical treatment completion. Tafenoquine efficacy and non-inferiority compared with primaquine in non-G6PD-deficient patients has been demonstrated in several studies, but this molecule is not yet available in France [26, 29]. These results are made even more relevant by the unexpected sharp increase in new malaria cases on the Guiana Shield, particularly in the Brazilian state of Amapá and French Guiana, in 2023–2024 [30].

**Fig. 6:**
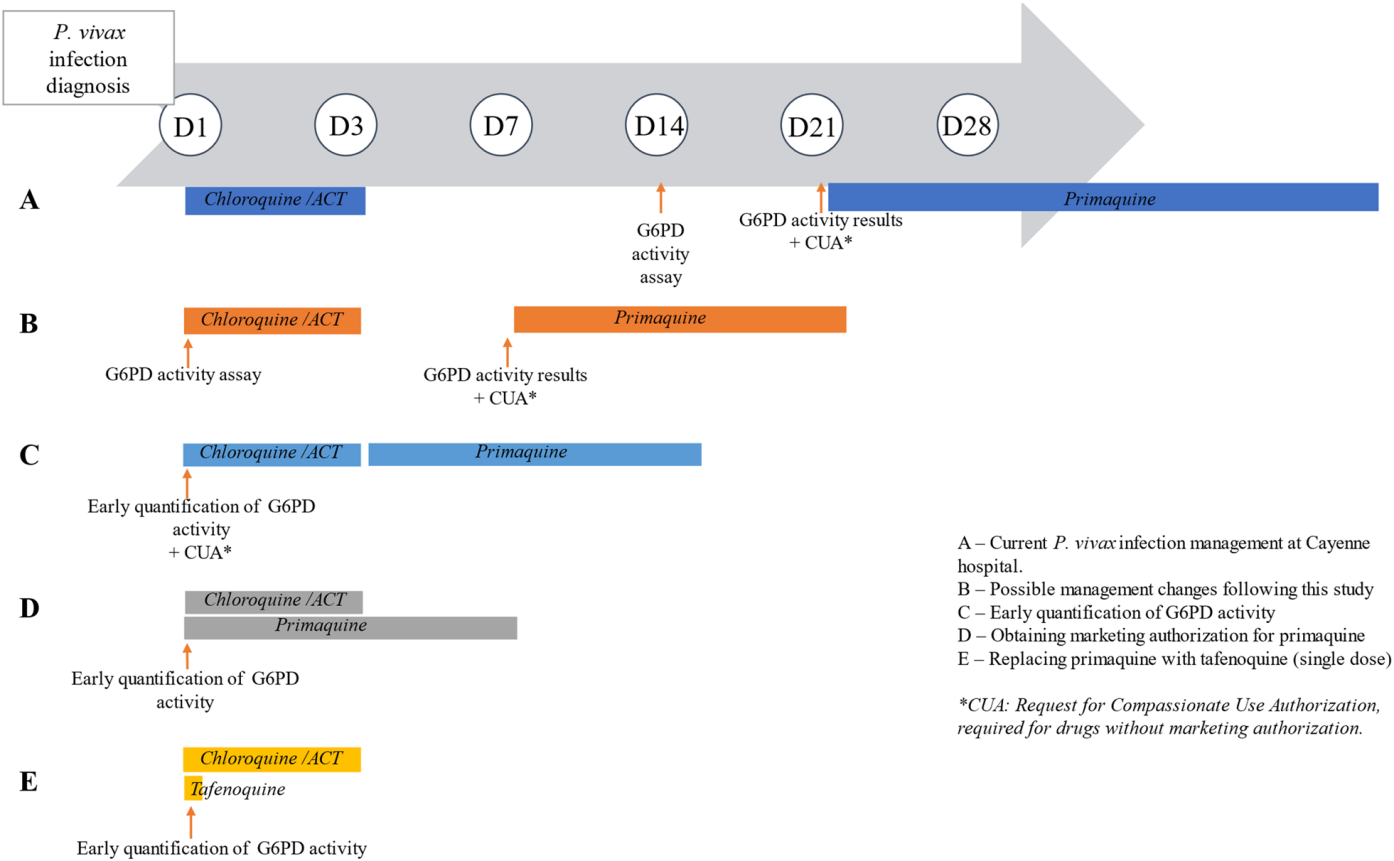
Optimization of *P. vivax* infection management using different diagnostic and treatment levers

Optimization of radical treatment for *P. vivax* infections is essential to reduce parasite transmission and malaria eradication. Early detection of G6PD deficiency and prompt delivery of primaquine appear to be possible levers of action. Thus, the development and validation of a tool for the rapid quantitative assessment of G6PD activity is essential. It would drastically shorten the G6PD deficiency screening time and the interval before radical treatment initiation (Fig. 6) [31–33].

Lastly, several studies suggest a synergy between primaquine and chloroquine [34–37]. Therefore, the implementation of rapid G6PD assays with early delivery of primaquine, concomitantly with chloroquine treatment, could benefit the patient. This remains a hypothesis that needs to be confirmed.

## Conclusion

This pragmatic study highlights the absence of variability in G6PD activity over time in patients treated for *P. vivax* malaria infection in French Guiana. If these results are confirmed by larger studies, G6PD activity could be measured early to promptly initiate radical treatment in patients without G6PD deficiency, or repeat the measurement if there is a deficit in the initial phase. Considering the large number of early relapses and patients lost to follow-up before radical treatment, this change in management could have a major public health impact.

### Supplementary Information

Supplementary Material 1.

## Data Availability

Data are available from the Clinical Investigation Center, Inserm 1424, at the Cayenne Hospital.
