# Supplementary material for: Kinetics of glucose-6-phosphate dehydrogenase (G6PD) activity during Plasmodium vivax infection: implications for early radical malaria treatment
Source: Malar J. 2024 May 9;23:140. doi: 10.1186/s12936-024-04973-4 (PMC11080303; doi:10.1186/s12936-024-04973-4)
Supplement: Supplementary file 1 — Supplementary Material 1. [file 12936_2024_4973_MOESM1_ESM.docx]

# Supplementary material

Table of contents

[Supplementary material 1](#_Toc160098382)

[Supplement 1 : Raincloudplots of G6PD activity· from day 1 to day 28 after treatment initiation with chloroquine or artemisinin-based combination therapy. 5](#_Toc160098383)

[Supplement 2 : Spaghetti plot showing G6PD activity measurements in each individual patients from day 1 to day 28 after treatment initiation with chloroquine or artemisinin-based combination therapy.. Patients with complete data : day 1= 20, day 3= 51, day 7= 54, day 14= 61, day 21= 46, day 28= 52. Red line : G6PD activity = 30%. Red dashed line:G6PD activity = 80%. Red dash dotted lines: G6PD activity =10%. 9](#_Toc160098384)

[12](#_Toc160098385)

[Supplement 3: Spaghetti plot showing G6PD activity measurements in each male patient to describeg its individual variability during the follow-up (n=48 patients). D: day. 12](#_Toc160098386)

[13](#_Toc160098387)

[Supplement 4: Spaghetti plot of G6PD activity measurements in each female patient to describe its individual variability during the follow-up (n=45 patients). D= day. 13](#_Toc160098388)

[Supplement 6: Prevalence of G6PD deficiency over time. 16](#_Toc160098389)


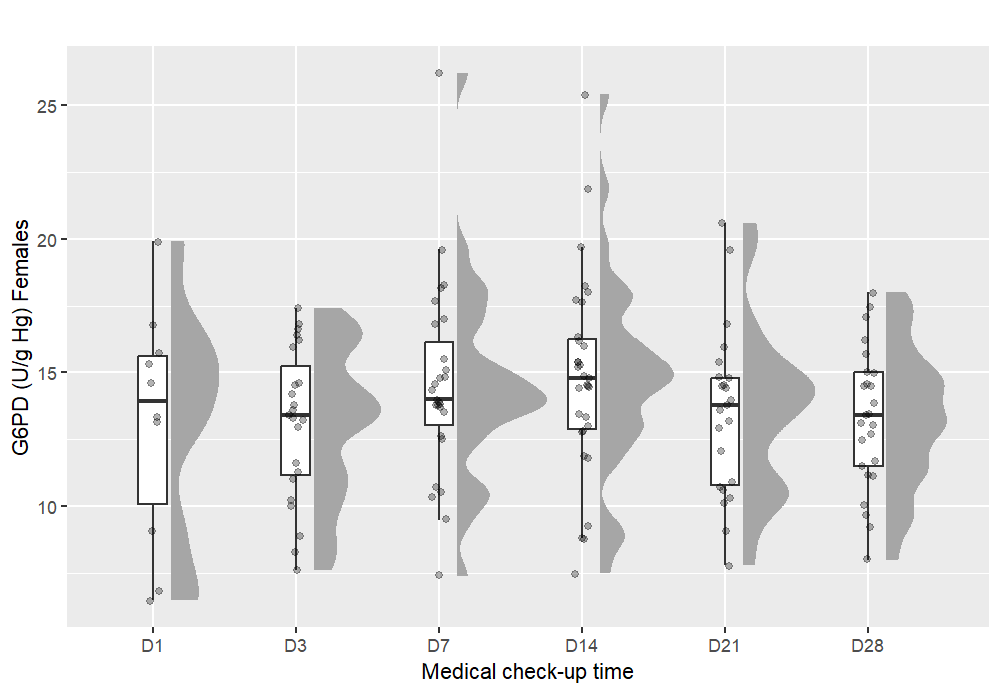

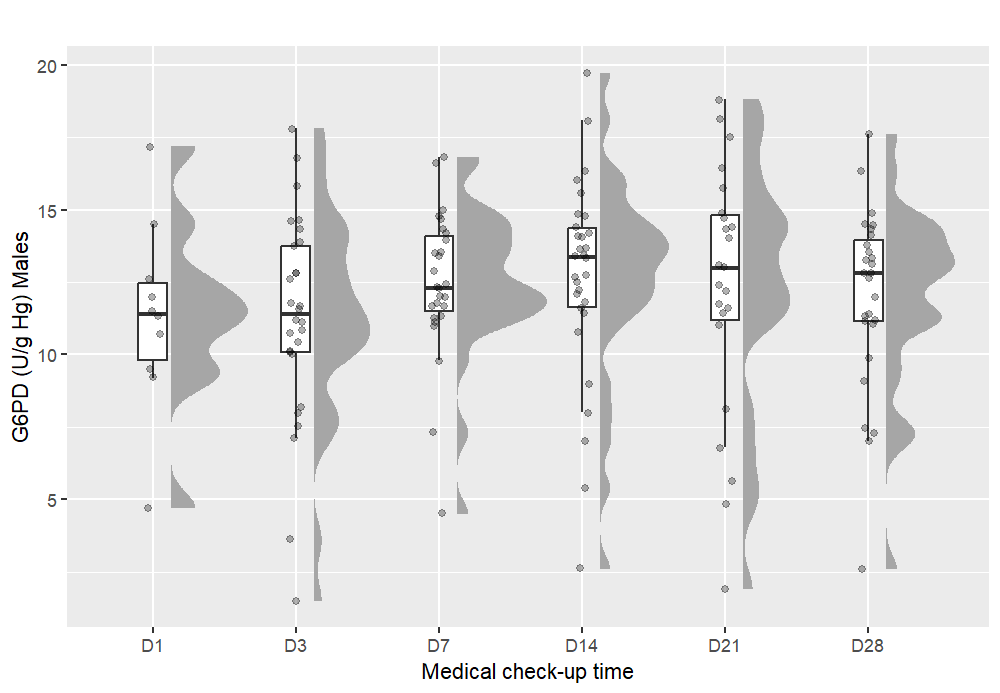


##### Supplement 1: Raincloudplots of G6PD activity· from day 1 to day 28 after treatment initiation with chloroquine or artemisinin-based combination therapy.


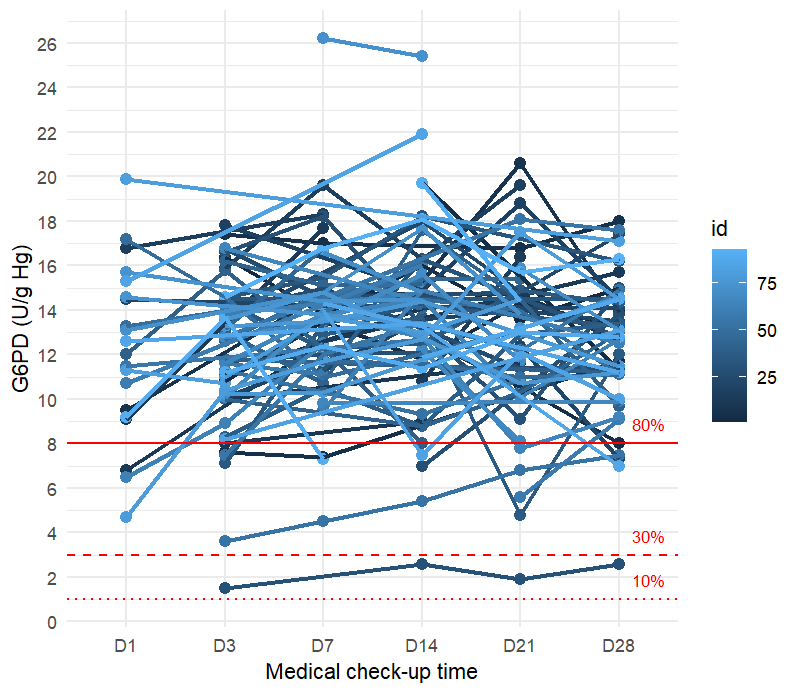


##### Supplement 2: Spaghetti plot showing G6PD activity measurements in each individual patients from day 1 to day 28 after treatment initiation with chloroquine or artemisinin-based combination therapy.. Patients with complete data : day 1= 20, day 3= 51, day 7= 54, day 14= 61, day 21= 46, day 28= 52. Red line : G6PD activity = 30%. Red dashed line:G6PD activity = 80%. Red dash dotted lines: G6PD activity =10%.

D1 D3 D7 D14 D21 D28

Time

#####
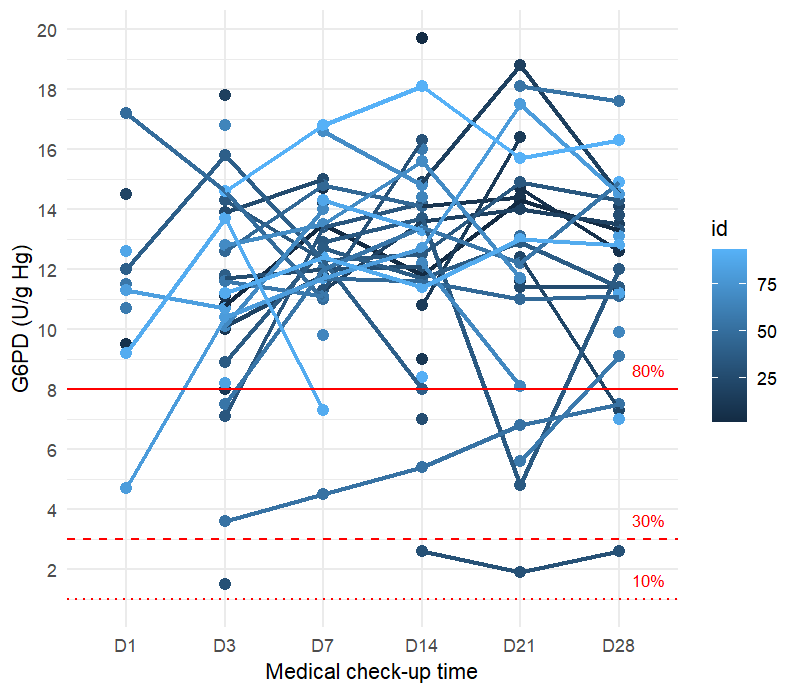


##### Supplement 3: Spaghetti plot showing G6PD activity measurements in each male patient to describeg its individual variability during the follow-up (n=48 patients). D: day.

D1 D3 D7 D14 D21 D28

Time

#####
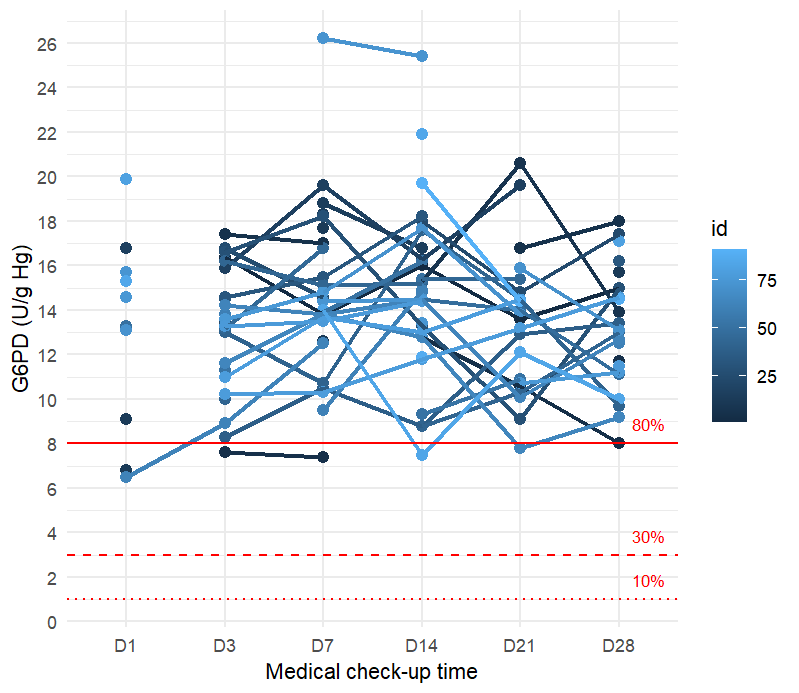


##### Supplement 4: Spaghetti plot of G6PD activity measurements in each female patient to describe its individual variability during the follow-up (n=45 patients). D= day.

**Supplement 5 :** **Correlation of the fixed effects in the model presented in Table 4.**

| Intercept | Intercept | Day-3 | Day-7 | Day-14 | Day-21 | Day-28 | Sex |
| --- | --- | --- | --- | --- | --- | --- | --- |
| Day-3 | 0.356 | **--** | **--** | **--** | **--** | **--** | **--** |
| Day-7 | -0.370 | -0.665 | **--** | **--** | **--** | **--** | **--** |
| Day-14 | -0.078 | 0.552 | -0.453 | **--** | **--** | **--** | **--** |
| Day-21 | 0.300 | -0.227 | 0.84 | -0.229 | **--** | **--** | **--** |
| Day-28 | -0.350 | -0.023 | -0.070 | 0.281 | 0.027 | **--** | **--** |
| Sex | -0.51 | -0.041 | 0.066 | -0.032 | 0.015 | -0.007 | **--** |
| Ln (reticulocyte) | -0.978 | 0.451 | 0.451 | 0.018 | -0.281 | 0.332 | 0.024 |

|  |  | G6PD activity day 1 |  |  | G6PD activity day 3 |  |  | G6PD activity day 7 |  |
| --- | --- | --- | --- | --- | --- | --- | --- | --- | --- |
|  | **Males** | **Females** | **Total** | **Males** | **Females** | **Total** | **Males** | **Females** | **Total** |
| <10% | 0/24 (0) | 0/13 (0) | 0/37 (0) | 0/38 (0) | 0/24 (0) | 0/62 (0) | 0/37 (0) | 0/31 (0) | 0/68 (0) |
| 10-30% | 1/24 (4·2) | 0/13 (0) | 1/37 (2·7) | 3/38 (7·9) | 0/24 (0) | 3/62 (4·8) | 0/37 (0) | 0/31 (0) | 0/68 (0) |
| 30-80% | 3/24 (12·5) | 2/13 (15·4) | 5/37 (13·5) | 4/38 (10·5) | 1/24 (4·2) | 5/62 (8·1) | 2/37 (5·4) | 1/31 (3·2) | 3/68 (4·4) |
| >80% | 20/24 (83·3) | 11/13 (84·6) | 31/37 (83·8) | 31/38 (81·6) | 23/24 (95·8) | 54/62 (87·1) | 35/37 (94·6) | 30/31 (96·8) | 65/68 (95·6) |
| Missing data | 108/132 | 78/91 | 186/223 | 94/132 | 67/91 | 161/223 | 95/132 | 60/91 | 155/223 |

|  |  | G6PD activity day 14 |  |  | G6PD activity day 21 |  |  | G6PD activity day 28 |  |
| --- | --- | --- | --- | --- | --- | --- | --- | --- | --- |
|  | **Males** | **Females** | **Total** | **Males** | **Females** | **Total** | **Males** | **Females** | **Total** |
| <10% | 0/61 (0) | 0/52 (0) | 0/113 (0) | 0/38 (0) | 0/31 (0) | 0/69 (0) | 0/37 (0) | 0/34 (0) | 0/71 (0) |
| 10-30% | 1/61 (1·6) | 0/52 (0) | 1/113 (0·9) | 1/38 (2·6) | 0/31 (0) | 1/69 (1·5) | 1/37 (2·7) | 0/34 (0) | 1/71 (1·4) |
| 30-80% | 4/61 (6·6) | 3/52 (5·8) | 7/113 (6·2) | 3/38 (7·9) | 1/31 (3·2) | 4/69 (5·8) | 3/37 (8·1) | 0/34 (0) | 3/71 (4·2) |
| >80% | 56/61 (91·8) | 49/52 (94·2) | 105/113 (92·9) | 34/38 (89·5) | 30/31 (96·8) | 64/69 (92·7) | 33/37 (89·2) | 34/34 (100) | 67/71 (94·4) |
| Missing data | 71/132 | 39/91 | 110/223 | 94/132 | 60/91 | 154/223 | 95/132 | 57/91 | 152/223 |

##### Supplement 6: Prevalence of G6PD deficiency over time.
